# Evaluation of Nociception during Pediatric Surgery: A Topical Review

**DOI:** 10.3390/jpm13020260

**Published:** 2023-01-30

**Authors:** Gianluca Bertolizio, Marta Garbin, Pablo M. Ingelmo

**Affiliations:** 1 Department of Pediatric Anesthesiology, Montreal Children’s Hospital, Montreal, QC H4A 3J1, Canada; 2 Department of Anesthesia, Faculty of Medicine and Health Sciences, McGill University, Montreal, QC H4A 3J1, Canada; 3 Research Institute, McGill University Health Center, Montreal, QC H4A 3J1, Canada; 4 Department of Clinical Sciences, Université de Montréal, St-Hyacinthe, QC J2S 2M2, Canada; 5 Edwards Family Interdisciplinary Center for Complex Pain, Montreal Children’s Hospital, Montreal, QC H4A 3J1, Canada; 6 Alan Edwards Center for Research on Pain, McGill University, Montreal, QC H3A 2B4, Canada

**Keywords:** analgesia, ANI, children, electroencephalography, galvanic skin response, heart rate variability, NIPE, nociception index, nociceptive flexion reflex, plethysmography, pupillometry, skin conductance, surgical pleth index

## Abstract

The association between intraoperative nociception and increased patient’s morbidity is well established. However, hemodynamic parameters, such as heart rate and blood pressure, may result in an inadequate monitor of nociception during surgery. Over the last two decades, different devices have been marketed to “reliably” detect intraoperative nociception. Since the direct measure of nociception is impractical during surgery, these monitors measures nociception surrogates such as sympathetic and parasympathetic nervous systems responses (heart rate variability, pupillometry, skin conductance), electroencephalographic changes, and muscular reflex arc. Each monitor carries its own advantages and disadvantages. The manuscript aims to give an overview of the most up-to-date information available in the literature on current nociceptor monitors available in clinical practice, with particular focus on their applications in pediatrics.

## 1. Introduction

Perioperative pain carries significant morbidity, including cardiovascular complications, immunosuppression, and development of chronic pain [1]. Intraoperative nociceptive stimuli are still processed centrally even in presence of deep general anesthesia [2], therefore, optimal nociception control is pivotal in anesthesia practice, as advocated by the Safetots initiative [3]. Monitoring of intraoperative nociception (neural encoding and the processing of noxious stimuli without conscious perception) has gained popularity in the recent years.

An ideal nociception monitoring would optimize the administration of intraoperative analgesics and reduce overdose side effects, infer the quality of intraoperative regional blocks, and avoid unnecessary oversedation [4]. The collection of intraoperative data of the nociceptive response would also promote research on the interaction between intraoperative nociception and postoperative outcomes [4].

The monitoring and modulation of intraoperative nociception represents a big challenge for researchers and clinicians. First, the nociceptive ascending pathways or subcortical centers can be evaluated only through complex investigations, such as functional magnetic resonance imaging [4], which is clearly unfeasible in the daily practice. Second, there are various classes of nociceptors, which respond to temperature, pressure, chemical and tissue damage stimuli [5]. Nociceptors are characterized by an all-or-none response, which is transmitted on different speeds by axons of different size and myelinization [5]. As a results, nociception pathways may not all be blocked by a single analgesic agent, such as opioids [6]. Third, there is no gold standard for intraoperative nociception monitoring, which makes the validation of any new modality to measure nociception difficult. Finally, data in pediatrics are limited, and any extrapolation from the adult literature remains artificial.

## 2. Nociception Monitors

The nociception monitors employable during surgery are listed in Table 1.

### 2.1. Somatic and Autonomic Responses Monitoring

Noxious stimulus during surgery leads to a peripheral autonomic response that results in lacrimation, patient movements, tachypnea, tachycardia, and hypertension [4]. Patient’s movements, tachycardia, and hypertension are still the most common parameters used to guesstimate the level of intraoperative analgesia, and are often used as reference to validate emerging analgesia monitors [7]. However, these responses can be affected by factors not related to nociception, such as medications (i.e., muscle relaxants, beta blockers) and medical conditions (i.e., heart transplant, pacemaker, hypovolemia).

Heart rate and blood pressure poorly correlate with brain and spinal cord nociception [2]. In children under general anesthesia, maximal tetanic stimulation may lead to an increase in heart rate of only 5% [8]. Therefore, hemodynamic changes are not always and uniquely related to intraoperative nociception. When variations in heart rate and blood pressure occur, the administration of analgesic (opioids) may be appropriate (the dose is proportional to a nociceptive stimulus), inappropriate (the dose is insufficient or excessive in comparison to a nociceptive stimulus), or unnecessary (the hemodynamic changes are not secondary to a nociceptive stimulus). Consequently, the use of heart rate and blood pressure as markers of adequate analgesia has led to intraoperative opioids overuse and possible related side effects such as postoperative hyperalgesia [6].

### 2.2. CARDEAN Index

Heart rate and non-invasive blood pressure have been integrated in the CARDEAN Index (CARdiovascular DEpth of ANalgesia, Alpha2, Lyon, France) to monitor nociception during surgery [9]. On a 100-point scale, values >60 correspond to the somato-sympathetic reflex (hypertension and tachycardia) whereas values ≤60 represent the vagal baroreflex (hypertension and bradycardia) [6,9]. In the presence of adequate hypnosis, the use of CARDEAN index has been associated with a decreased incidence of intraoperative tachycardia and opioid use [6].

Recently, the CARDEAN index has been integrated in the Philips Intellivue^®^ monitor [6]. The CARDEAN index may be affected by arrhythmias and inotropic, chronotropic, and vasoactive drugs [9]. To date, no data in children are available.

### 2.3. Electroencephalogram (EEG)

Electroencephalogram (EEG) signal has been integrated in various nociception monitors such as the Brain Anaesthesia Response monitor (BAR, the Cortical Dynamics Ltd., North Perth, Australia), the qNOX (Quantium Medical S.L., Barcelona, Spain), and the Spectral Entropy (GE Healthcare, Helsinki, Finland) [9,10]. Common EEG intraoperative monitors group wave signals in four frequency bands: <4 Hz (delta), 4–8 Hz (theta), 8–13 Hz (alpha),13–25 Hz (beta) and 25–40 Hz (gamma) [11]. These clusters have been used to develop specific indexes and Density Spectral Array (DSA) outputs [12].

During general anesthesia, a noxious stimulus can trigger the beta arousal (increased power in the beta-frequency band), delta arousal (increased power in the delta band) and alpha dropout (decreased alpha power) [10,13]. Beta arousals typically occur during light anesthesia, contrary to delta arousals and alpha dropouts that occur during a deep anesthesia. In the EEG spectrogram these three events are visualized as: (1) an increase of warm colors (power) in the beta range, (2) an intensification of warm colors in the delta range and (3) a sudden, temporary turn from warm to cooler colors in the alpha range [10].

The main limitation of EEG is represented by its interpretation in the context of general anesthesia: while a beta arousal can be seen as a trend toward patient awakening, delta arousal and alpha dropout may be misclassified as an excessive level of hypnosis [10]. Moreover, patient’s conditions such as neurodegeneration, stroke, age, cognitive impairments alter the baseline EEG waves [10]. Intraoperative EEG has shown limitations in monitoring anesthesia depth of small children and particularly infants [12]. In fact, EEG indexes lack of a linear relationship with sevoflurane concentration, are poorly reliable when ketamine or nitrous oxide are employed, and do not correlate with the depth of the anesthesia in children less than 3 years old [10,11,12]. Moreover, DSA have shown drugs specific patterns that make the interpretation of the anesthesia depth challenging [12]. These limitations may explain the lack of intraoperative EEG data on pediatric nociception monitoring.

### 2.4. Functional Near-Infrared Spectroscopy (fNIRS)

Near infrared spectroscopy (NIRS) continously measures regional tissue oxigenated and de-oxyganated hemoglobin. Initially developed to monitor oxygen uptake/consumtpion of the brain, NIRS has been applied to other tissues such as kidney and has been widely used in pediatric open-heart surgery and intensive care [14]. Functional NIRS (fNIRS) measures changes in the hemoglobin oxygenation (oxygenated and de-oxygenated) as a function of cerebral activity [15]. The technology has recently been employed to investigate nociception-related brain activity in a number of diseases and conditions [16]. Changes of 0.3 mM of oxygenated hemoglobin in specific regions of the brain (i.e., somatosensory and frontopolar cortexes) have been associated to intraoperative nociceptive events [17]. Data on fNIRS and nociception are still preliminary and limited to experimental data in adults.

Motion artifacts, noise interference, hemodynamic changes not related to cerebral activity, need of multiple optical sensors and challenges in cerebral mapping currently represent the major limitations in fNIRS [16], despite the rapid evolution of this technology.

### 2.5. Spectral Entropy

Spectral entropy monitor analyzes the EEG entropy (i.e., the degree of perturbation or randomness) and the electromyography (EMG) signal to calculate the response entropy (RE) and the state entropy (SE) as a measure of intraoperative analgesia [12,18,19]. The RE is computed from a frequency range of 0.8–47 Hz and integrates both EEG and EMG signals, whereas the SE derives from an EEG frequency range of 0.8–32 Hz and represents the depth of hypnosis [18]. A difference between the two (ΔRE-SE) less than 10 was associated with a decrease of intraoperative opioid administration [9,18]. Whether the ΔRE-SE simply measures the level of (in)adequate anesthesia rather than nociception is still unclear. This is probably why studies on ΔRE-SE are lacking in both adults and children.

### 2.6. qNOX Index

The qNOX index is one component of the CONOX^®^ monitor (Fresenius Kabi, Brézins, France), which is based on the integration of an artificial neural network with a fuzzy logic system [20,21]. The qNOX index was developed from a Ramsay scale 5 and 6 as reference and integrated with the qCON component (developed from EEG data) [20]. The qNOX uses raw EEG and EMG signals to predict the likelihood of a response to nociception. The score is displayed on a 100-point scale (0–99), being values >60 indicative of high likelihood of nociception in adults [21,22]. A recent investigation, however, showed that qNOX correlated poorly (*r* = 0.3) with the intraoperative remifentanil infusion rate and the Analgesia Nociception Index (ANI) values [21]. No data exists in children.

### 2.7. Nociceptive Flexor Reflex

The electrical intensity needed to elicit a spinal polysynaptic withdrawal reflex can be used as surrogate of the level of nociception [18]. A clinically adequate, opioid-based general anesthesia (absence of somatic and hemodynamic responses to high intensity tetanic stimulations) abolishes only 59% of the spinal cord and brain nociception, which is still detectable with functional magnetic resonance imaging [2].

The Nociceptive flexor reflex (NFR or RIII reflex, Dolosys GmbH, Berlin, Germany) measures the EMG activity secondary to the electrical stimulus (on the biceps femoris muscle) and has been studied during general anesthesia [18,23]. Current data show a 63% probability of predicting nociception [22]. However, age, gender, weight, neuromuscular blockers, and muscular diseases limit its application [18]. Data in pediatrics are not available.

### 2.8. Newborn Infant Parasympathetic Evaluation (NIPE) and Analgesia Nociception Index (ANI)

In recent years, investigations have focused on parasympathetic tone activity as an indirect assessment of the level of nociception. While heart rate variability (HRV) is affected by the balance between sympathetic and parasympathetic tone, the high frequency changes of HRV are primarily mediated by and specific to the parasympathetic nervous system [24,25,26]. The phenomena of pain, fear, anxiety, and intraoperative nociception have proven to be accompanied by changes in HRV [27,28,29,30]. In pediatric patients, HRV analysis correlates with prolonged pain [31,32] and newborn discomfort [33,34,35].

The newborn infant parasympathetic evaluation (NIPE, MDoloris Medical Systems, Loos, France) is a non-invasive, standardized continuous measurement of HRV. The cardiac signal is extrapolated from the electrocardiogram, and a wavelet based high pass filter over 0.15 Hz is applied in order to keep parasympathetic related variations [36]. The NIPE monitor displays two averaged measurements: the average NIPE (NIPEa) results from the average of NIPE measured over the previous 20 min, and the current NIPE (NIPEc) is calculated on a 64-s sliding window. An algorithm [37] derived from the high frequency changes of the HRV calculates a score between 0 and 100, where a score of 0 indicates minimal parasympathetic tone and maximal nociception or discomfort. Non-anesthetized infants undergoing procedural pain (heel pick) showed a median NIPEc max values of 52.5 (43.0–59.0) and 50.0 (44.5–59.0) for no/mild/moderate and severe pain, respectively [38]. In anesthetized children <2 years a NIPE < 50 may guide opioid administration [39]. More recently, a 15–30% decrease in NIPE values has shown a sensitivity of 92% and a specificity of 96% for nociception during venous puncture, surgical skin incision and intubation [39]. In 2-year-old children undergoing brief tetanic stimulations (5 s, 100 Hz, 10 to 60 mA), NIPE proportionally decreased from a baseline of 75 ± 10 to 48 ± 12 (60 mA), whereas heart rate changed minimally [40].

The NIPE index is a modification of the ANI (Mdoloris Medical Systems, Loos, France) and was developed for infants and young children who have baseline heart rate higher than adults, resulting in a possible lower variability. Similar to NIPE, ANI expresses the relative amount of parasympathetic tone present as compared to sum of sympathetic and parasympathetic activities. The ANI Monitor displays two averaged ANI measurements: the ANIi results from the average of ANI measured over the previous 120 s, and the ANIm results from the average of ANI measured over the previous 240 s. The ANI algorithm is set for a heart rate range of 30–180 beats/min, whereas the NIPE algorithm range is between 80 and 250 beats/min. The ANI has shown a good performance in predicting intraoperative nociceptive stimuli in animals [41], adults [31,41] and older children [8,42,43,44,45]. In adults, ANI showed 88% sensitivity and 83% specificity in predicting hemodynamic changes [22]. In children, sensitivity and specificity of ANI values ≤50 to predict intraoperative nociception (increased heart rate by 10% during skin incision) were 79% and 62%, respectively [42]. However, it has been reported that 30–50% of patients may lie in the inconclusive zone [43], suggesting that further studies are warranted.

Patient’s conditions such as arrhythmias or pacemakers and medications such as alpha_2_- or beta_1_-adrenergic agonists and antimuscarinics can affect ANI and NIPE values [46].

### 2.9. Pupillometry

The pupillary radial muscle has a sympathetic innervation and causes pupillary dilatation, whereas the circular muscle has a parasympathetic innervation and causes pupillary constriction [47,48]. After a nociceptive stimulus, the sympathetic-mediated pupillary reflex dilation begins in 3 s and peaks within a minute [47].

Pupillometry has been marketed by several companies (i.e., ANeurOptics PLR-100, NeurOptics, Laguna Hills, CA, USA; Algiscan system, IDMed, Marseille, France) [18]. Several indexes can be extrapolated, such as the neurologic pupillary index, the constriction velocity, the dilatation velocity, the Pupillary Reflex Dilatation (PRD) and the Pupillary Pain Index (PPI).

A PRD amplitude between 13% and 25% from baseline is considered indicative of nociception in absence of hemodynamic response [48]. In children aged 2–15 years, PRD variations were more sensitive to surgical skin incision than hemodynamic changes, increasing by 160–200% contrary to 7–10% of heart rate and 5–8% of systolic blood pressure [49]. In children with burn injuries, aged 1–13 years and anesthetized with ketamine [50], the pupillary diameter increased linearly with the incrementation of the tetanic stimulations to a maximal mean dilation of 39% (±19%). It must be noted that in above studies, the baseline pupillary diameter varied from 2.3 to 3.4 mm, which may explain the variability in the PRD response (i.e., higher basal pupillary diameter, lower maximal possible variation). A PRD amplitude above 32% showed a 65% sensitivity and 77% specificity for movement response to nociception. In children aged 3–12 years, PRD-guided analgesia (PRD between 5–30%) was associated with a 25% decrease in remifentanil consumption compared to blood pressure-guided analgesia (defined as changes of ±20% from the baseline) [51]. In a study on children aged 1–16 years in which an increased heart rate by 10% was used as marker of nociception, a pupillary diameter cut-off value of 4.2 mm showed a sensitivity of 58% and specificity of 79% [42]. In the same investigation, the PRD had a slight smaller area under the curve (AUC) compared with an ANI < 51 (0.67 versus 0.75, respectively), which showed in a sensitivity of 79% and specificity of 62%. In children above 2 years of age, an increased PRD of 0.2 mm above the baseline was suggested as threshold of nociceptive response [48]. In this investigation, children >2 years showed a greater maximum PRD (1.3 mm, range 0.3–2.6 mm) than younger children (0.6 mm, range 0.3–1.6 mm).

The PPI can be measured through an infrared videopupillometer applied over the orbit with the aid of an opaque silicon cylinder [47] while a tetanic stimulation (200-microseconds, 100 Hz) is delivered on the patient ulnar nerve. From a starting intensity of 10 mA, each step consists in stimulations increased by 10 mA up to 60 mA, after which the intensity remains constant and the duration is prolonged by 1 s for a maximum of 3 s [52,53]. An PRD increase of 13% determined the PPI [52,53]. The PPI ranges from 1 to 10, being 10 maximal pupillary reactivity [47], and a PPI > 7 suggests insufficient analgesia [18]. In both adults and children, a PPI in a range of 0.5–3 is associated with a reduction of intraoperative nociception and opioid consumption [47]. In children >2 years without surgical stimulation, PPI decreased by 3 points (95% CI from −4 to −2) after a bolus of 10 mcg/kg of sufentanil [52]. In presence of surgical stimulation (skin incision), PPI only showed a modest positive correlation with heart rate and PRD (*r* of 0.35 and 0.54, respectively) [53]. A pre-incision PPI cut-off of 3 showed a sensitivity of 80% and a specificity of 79% in predicting the absence of heart rate changes to nociception [53].

Based on data present in literature, pupillometry appears to be helpful in detecting intraoperative nociceptive stimuli. Data are less convincing in children <2 years, probably due to the incomplete maturation of the optic nerve which may blunt the pupillary response [47,48].

The main disadvantages of the pupillometry consist in the bulk of the system (which limits its utilization in certain surgeries) and the need of individual calibration before the nociceptive stimulus (generally before surgical incision) [47]. Medications such as neostigmine, droperidol, metoclopramide and clonidine as well as pupillary diseases may affect the measurements [7,48]. On the contrary, esmolol and ondansetron do not [7]. Finally, opioids, but not inhalational or intravenous anesthetics, affect the PRD [48].

### 2.10. Skin Conductance

The Skin Conductance Algesimeter (SCA, MedStorm innovations, AS, Oslo, Norway) aims to measure the skin conductance caused by rapid micro-fluctuations of water permeability at the level of the palms (or soles), as the sweat glands in these regions are exclusively innervate by the sympathetic system [22,47]. Nociception, by increasing the sympathetic tone (i.e., increasing sweat), leads to an increase of frequency and amplitude of the skin conductance [47].

Skin conductance is measured as number of skin conductance fluctuations per second (NFSC) [22], which have the advantage of fast response (<2 s) and short duration (<0.7 s) [54]. In adults, it correlates with intraoperative blood pressure and plasma catecholamines concentrations, but not with opioid administration [47]. As per manufacturer recommendation, NFSC within 0–0.07 corresponds to no pain, NFSC within 0.13–0.21 corresponds to a Visual Analogue Scale (VAS) less than 40, NFSC 0.21–0.26 to VAS of 40–50, NFSC 0.26–0.33 to a VAS of 60–80, and NFSC of 0.40–0.7 to a VAS of 80–100 [18].

In 20 ventilated intensive care children sedated with different protocols and undergoing tracheal suctioning, changes in the COMFORT scale could be predicted by NFCS in about 50% of the cases (46–61%). After tracheal stimulation, however, NFCS did not change significantly compared with baseline [0.00 (range 0.00–0.14) versus 0.00 (range 0.00–0.03]), respectively] [54].

A pilot investigation on 36 infants <3 months of age [55] compared the skin conductance, measured as peak per seconds (PPS, equivalent to the NFCS), with NIPE in response to heel stick. NIPE and PPS changed from 50.5 (range 44.0–59.0) and 0.00 (range 0.00–0.14) before the procedure to 42 (range 35.5–47.0) and 0.60 (range 0.47–0.73) after stimulation, respectively. Despite a significative difference between pre-and post-stimulation values, the two monitors were unable to differentiate patients with moderate/or less pain from those with severe pain. Of notice, 12% of the patients had unreliable NIPE and PPS data due to motion artifacts. Despite some limitations (i.e., small sample size, different pain scale employed in relation to the patient age), this study highlighted the narrower variability in baseline skin conductance (0.00–0.14) compared with NIPE (44.0–59.0), suggesting that nociception might be better detected by small variations of skin conductance than NIPE.

These findings were not confirmed intraoperatively by the only pilot study available in the literature [8]. NFSC, ANI, heart rate and blood pressure responses were investigated in relation to tetanic stimulation (5 s, 50 Hz, 50 mA) and changes in remifentanil infusion rate in 12 children 3–15 years old, maintained at a constant depth of anesthesia (Bispectral Index of 50) [8]. In absence of nociceptive stimulation, both NFSC and ANI did not vary regardless the remifentanil infusion rate. During stimulation, NFSC did not change significantly, albeit a positive trend was observed when the remifentanil rate was reduced, whereas ANI decreased proportionally to the remifentanil rate. Of note, when similar nociceptive conditions were applied, heart rate raised of only 5% while ANI decreased of 25%.

Skin conductance is not affected by temperature, hypoxia, volemia, and medications [56,57]. In children, it may be influenced by the depth of the anesthesia (reduced sympathetic activity) [8]. Despite the theorical advantages, data in pediatric are too scarce to make any recommendation.

### 2.11. Surgical Pleth Index (SPI)

Similar to palmar sweat glands, distal arterioles exclusively contain sympathetic alpha_1_ receptors [47]. The nociception-induced vasoconstriction results in a change of blood flow wave amplitude that can be measured by photoplethysmography [47]. The Surgical Pleth Index (SPI, GE healthcare, Helsinki, Finland) derives from the former Surgical Stress Index (SSI). The SPI combines the normalized photoplethysmography wave amplitude (PPGA_norm_) and the normalized heartbeat interval (HBI_norm_) into an algorithm that displays SPI values on a 100-point scale, being 100 maximum nociception [46,47]. The SPI is calculated according to the formula:

100 − (0.7 × PPGA_norm_ + 0.3 × HBI_norm_). (1)

In adults, intervals between 20 and 50 were associated with hemodynamic stability and faster extubation [47]. However, studies investigating the capability of SPI to decrease intraoperative opioid administration and hormone response showed contradictory results [18,47].

Children have different heart rate interval and vascular compliance compared to adults, which affect PPGA and HBI signals [58]. As a result, a SPI of 40 or lower may be a better cut-off in this population [58]. In a study involving children >4 years of age [59], the median SPI (named SSI in the study) was higher than baseline and above 50 (range 31.7–60.1) during intubation and surgical stimulation, but the SPI variability was high (ΔSPI range −9.9 to 37.5 after intubation and −7.2 to 16.9 after the beginning of surgery). Although the SPI increase was statistically significant, baseline SPI could be as low as 22.6 and as high as 58.0 and the study did not consider the dependency between observations. Therefore, data appear of little clinical relevance. In another investigation enrolling 58 children 2–12 years old, SPI-guided anesthesia (target SPI value <50) resulted in reduced fentanyl consumption, similar episodes of tachycardia, but a 11.2% higher incidence of hypertension compared to the control group [60]. In children of 3–19 years receiving sufentanil at different infusion rates [61], baseline SPI was always above 50 and did not change significantly (range 43.2–61.9) during cranial pinning despite an increase of 20% in blood pressure. In children <2 years underdoing inguinal hernia repair, SPI had high individual variability (range 37–67) and average values remain above 49 regardless the presence of noxious stimuli [62]. In particular, median SPI increased from 56 (IQR 45–67) to 78 (67–84) during surgical incision, but this increase was blunted in patient receiving the ilioinguinal/iliohypogastric block. In addition, intraoperative opioid administration was at the anesthesiologists’ discretion and the same number of patients (40%) received a bolus of fentanyl in response to a reaction to operation, making these results of difficult interpretation.

Intravascular volume status, antihypertensives, atropine, posture, and hypothermia can affect the SPI [46]. In children, data on SPI are lacking and appropriate pediatric algorithm or threshold are yet to be determined [47,58].

### 2.12. Nociception Level (NoL) Index

Photoplethysmographic pulse wave, galvanic skin conductance, accelerometer, and peripheral temperature have been integrated in the Nociception Level Index (NoL, Medasense, Ramat Gan, Israel) [47]. From these four parameters, a number of derivates are extrapolated, namely the photoplethysmographic waveform amplitude (PPGA), high frequency band HRV (HRV-HF) power, number of skin conductance fluctuations (NSCF) and others [63]. Currently, NoL is the only 4-parameter monitor on the market.

HRV used the same band as ANI (at the 0.15 to 0.4 Hz band power), but it is calculated from the photoplethysmogram rather than the electrocardiogram trace. Signals are gathered in 5-s windows, and values between 10 and 25 are considered ideal for analgesia [18].

Data are currently available only in adults, in which NoL has shown to correlate with intraoperative nociception and opioids consumption [47]. NoL-driven anesthesia results in better hemodynamic control and 30% reduction of opioid consumption [18]. NoL appears to be more sensitive than heart rate and blood pressure monitoring in detecting intraoperative nociception and it is not affected by remifentanil [22]. Medication such as beta_1_- and alpha_2_-agonists are known to impair NoL reading [46].

So far, NoL has been not investigated in pediatrics, mainly because the apparatus resides in finger probe, which has not been designed for children. In comparison with adults, children have a different vascular elastance which affects the photoplethysmographic wave pattern and its derivates [64]. Without a proper validation, the use of current NoL algorithm may remain inaccurate in pediatrics.

## 3. Prediction of Postoperative Pain

Data on the capability of nociception monitors in predicting postoperative pain remains scarce and mostly in adults, in which cut-off values, clinically utility and effect on outcomes are still unclear [65].

The sensitivity and specificity of intraoperative SPI < 30 to predict postoperative moderate/severe pain was reported 68% and 57% [66], although these results were not confirmed by other investigations [67]. In children <16 years, a SPI < 40 at the end of the surgery appears to have a sensitivity of 76% and a specificity of 62% to predictive moderate/severe postoperative pain [68]. In the age groups 2–3 and 4–8 years, sensitivity and specificity increased to 100% and 73%, and to 100% and 52%, respectively [68]. This data confirmed a previous publication, where SPI-guided anesthesia (target SPI > 50) resulted in lower pain score and less rescue analgesia in children undergoing adenotonsillectomy [60].

In adults, ANI values <50 at the end of the surgery showed a sensitivity and a specificity of 86% for immediate postoperative moderate/severe pain [65] but a different investigation found that ANI-guided anesthesia did not reduce the incidence of postoperative pain and rescue analgesia [69]. In children, the optimal NIPE cut-off has not been determined yet, and the NIPE threshold varies in the literature. In children <2 years of age, a postoperative NIPE < 55 showed a sensitivity of 70% and a specificity of 72% in detecting moderate to severe pain [70]. Only one study investigated whether intraoperative values of NIPE < 50 correspond with higher postoperative/discomfort and found no statistically significant association [OR 4.89 (95% CI 0.05–643.5), *p* = 0.09] [39].

Pupillometry-guided anesthesia (target PRD < 30%) was associate with a 48% decrease in postoperative opioid requirements in adults [71]. In children, patients undergoing pupillometry-based anesthesia showed similar pain score than the conventional group, which however was <3/10 in both groups [51].

At a cut-off value of 0.1, intraoperative NFSC showed a sensitivity of 89% and specificity of 74% to predict postoperative moderate/severe pain in adults [72]. In a large study enrolling 165 postoperative children aged 1–16 years, a NFCS cut-off value of 0.13 showed a sensitivity of 90% and a specificity of 64% in detecting moderate/severe pain, with a better performance in older children (AUC 0.76 at 1–3 years versus 0.83 at 8–16 years) [73]. These results were not confirmed in a smaller study in children 7–17 years old, in which a NFCS cut-off point of 0.2 showed a sensitivity of 51.9% and a specificity of 71% in recognizing moderate or severe pain after surgery [74]. No studies have associated intraoperative NFSC with the prevalence of postoperative pain.

Similarly, a NoL-driven analgesia (target < 10 after the surgery begins) showed a negative predictive value of 83% for preventing severe postoperative pain [75] and resulted in lower postoperative pain although similar opioid consumption in adults [76].

Currently, there is no clear evidence about the value of the NFR reflex in predicting postoperative pain [22]. Similarly, qNOX failed to demonstrate any predictive value with postoperative pain [77].

## 4. Future Directions

The current research in anesthesia is moving toward an objective, reliable way to guide our analgesic management. The development of a reliable nociceptive monitors represents the natural consequence of this practice.

Contrary to adults, the cardiovascular system of children is less frequently affected by diseases or pharmacological therapies, which might lead the anesthesiologist to believe that variations of heart rate and blood pressure are more likely secondary to nociception. Yet, multicenter large morbidity investigations have highlighted that everyday practice in pediatric anesthesia is largely individual-based rather than evidence-based [78,79]. In this view, it appears obvious to seek a monitor that guides the anesthesiologist in the nociception/pain management.

In the authors’ opinion, the sympathetic/parasympathetic balance represents an acceptable, simple, surrogate of intraoperative nociception. On this regard, multiparametric monitors, by assessing this balance from different perspectives, may perform better than those with one or two parameters. To date, the most studied multiparametric monitor is NoL. The algorithm employed in NoL was based on adult data and uses some parameters, such as skin conductance, that showed conflicting result in children. Therefore, its use in pediatric without a proper validation, as for any monitor developed for adults, should be discouraged.

Future research should be focused in creating and validating algorithms in children, with particular attention to group aged <2 years and with the acknowledgement that one-fits-all algorithm will unlikely exist.

## 5. Conclusions

The ideal intraoperative nociception monitor should be easy to use, reliable and employable in any condition. To date, all the marketed monitors have shown some sort of limitations. For example, HRV-based monitors are affected by patient conditions and medications such as inotropes (often employed in small infants), and are not suitable for certain surgeries (i.e., cardiac). NFR and pupillometry are affected by medications and do not provide a continuous monitoring, whereas the EEG requires expert knowledge for its interpretation. Consequently, all monitors may have limited application in patients who probably would benefit the most, such as critical ill children or those undergoing complex surgeries.

As there is no consensus on the gold standard to assess intraoperative nociception, the comparison of these monitors may result artificial and subjective. Most of the monitors lack of clear thresholds and pediatric validation and have been used only in a research setting. Literature is still limited in pediatrics.

To date, pupillometry and ANI/NIPE might have some advantages over other monitors. Pupillometry may be less dependent on medications than ANI/NIPE. On the contrary, NIPE appears more user-friendly as it interfaces with the routine anesthesia monitor and displays values easy to interpret. ANI, however, requires specific cardiac electrodes to be applied on the patient’s chest. Both pupillometry and NIPE showed a good reliability in measuring nociception and are supported by a fairly strong literature. The only study that compared pupillometry with ANI, showed slightly higher sensitivity but lower specificity in the ANI monitor [42]. Finally, skin conductance failed to produce convincing results in children and did not perform as well as NIPE in detecting nociception [55].

Despite these limitations, the intraoperative nociception monitors appear to be helpful in guiding anesthetic managements, and their use should be encouraged in addition to other, less reliable forms of monitoring, such as heart rate and blood pressure.

## Figures and Tables

**Table 1:** Nociception monitors.

Nociception Monitor	Sources of Measurement	Threshold under General Anesthesia	Limitations	Reference Used under General Anesthesia for Testing
**Monitors with data available only in adults**				
CARDEAN Index	Heart rate Non-invasive blood pressure	>60 = somato-sympathetic reflex ≤60 = vagal baroreflex	Arrhythmia Inotropic, chronotropic drugs Vasoactive drugs	Heart rate Movement
Electroencephalography	Electroencephalography signals	Beta arousals Delta arousals Alpha dropouts	Signal interpretation	Noxious stimuli
fNIRS *	Hemoglobin oxygenation	0.3 mM	Limited data	Noxious stimuli
Spectral entropy	Electroencephalography Electromyography	(Response Entropy-State Entropy) < 10	Unclear whether it measures the level of anesthesia or nociception	Noxious stimuli
qNOX Index	Electroencephalography Electromyography	>60 = high likelihood of nociception (adults) <40 = very low likelihood of nociception (adults)	Neuromuscular blockers	Hemodynamic response
Nociceptive flexion reflex	Electromyography	>31.9 mA mild nociception (LMA insertion) >42.9 mA high nociception (skin incision)	Age Gender Weight Muscular diseases Neuromuscular blockers	Tetanic stimulation Movement Heart rate
NoL Index *	Accelerometer Photoplethysmography Skin conductance Temperature	10–25	Chronotropic drugs Vasoactive drugs	Hemodynamic response Opioid consumption Tetanic stimulation
**Monitors with data available in adults and children**				
ANI/NIPE *	Heart rate variability	>50	Arrhythmias Cardiac pacemaker Chronotropic drugs Vasoactive drugs	Hemodynamic response Opioid consumption
Pupillometry PRD/PPI *	Pupil diameter fluctuations Pupillary light reflex and pupil reflex dilation Amplitude in response to noxious stimuli	PRD amplitude < 25% (<30% in children) PPI > 7	Equipment Medications	Hemodynamic response Tetanic stimulation
Skin conductance	Conductance variation secondary to micro-fluctuations of water permeability	N. of skin conductance fluctuations/second (NFSC) > 0.2 (adults) Unclear in children	Decreased sympathetic activity (anesthesia depth)	Intraoperative blood pressure Plasma catecholamines
SPI *	Photoplethysmographic amplitude Photoplethysmographic pulse interval	20 and 50 (adults) Undetermined in children	Arrythmias Antiarrhythmics Cardiac pacemaker Chronotropic drugs	Hemodynamic response Opioid consumption Hormonal response

* ANI, analgesia nociception index; fNIRS, functional near-infrared spectroscopy; NIPE, newborn infant parasympathetic evaluation; NoL; nociception level; PPI, pupillary pain index, SPI; surgical plethysmographic index.
